# A physico-chemical survey of inland lakes and saline ponds: Christmas Island (Kiritimati) and Washington (Teraina) Islands, Republic of Kiribati

**DOI:** 10.1186/1746-1448-2-8

**Published:** 2006-07-03

**Authors:** Casey Saenger, Michael Miller, Rienk H Smittenberg, Julian P Sachs

**Affiliations:** 1 Massachusetts Institute of Technology, Department of Earth Atmospheric and Planetary Science, 77 Massachusetts Avenue, Building E34-205, Cambridge, MA 02139, USA; 2 University of Cincinnati, Department of Biology, Cincinnati, OH 45221, USA; 3 University of Washington, School of Oceanography, Box 355351, Seattle, WA 98195, USA

## Abstract

The equatorial Pacific Ocean atoll islands of Kiritimati and Teraina encompass great physical, chemical and biological variability within extreme lacustrine environments. Surveys of lake chemistry and sediments revealed both intra- and inter-island variability. A survey of more than 100 lakes on Kiritimati found salinities from nearly fresh to 150 ppt with the highest values occurring within the isolated, inland portions of the island away from the influence of groundwater or extreme tides. Dissolved oxygen (DO) and pH values also showed considerable variability with a less regular spatial pattern, but were both generally inversely related to salinity. Series of lakes, progressively more isolated from marine communication, present a modern analog to the chemical and morphologic evolution of presently isolated basins. Sediments on both islands consist of interbedded red and green silt, possibly degraded bacterial mat, overlying white, mineralogenic silt precipitate. Variability may be indicative of shifts in climatological parameters such as the El Niño Southern Oscillation (ENSO) or the Pacific Intertropical Convergence Zone (ITCZ).

## Background

**Figure 1:**
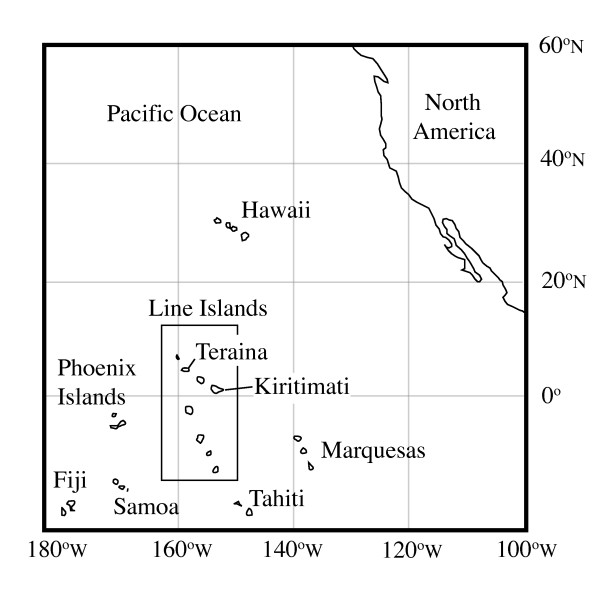
Map of the central North Pacific Ocean showing the location of Kiritimati and Teraina islands.

The equatorial Pacific coral atoll lakes and lagoons of Kiritimati (Christmas) and Teraina (Washington) Islands, Republic of Kiribati (Figure 1), possess a breadth of lacustrine environments from fresh to hypersaline in which a range of tropical limnologic conditions can be examined within a small geographic area. Previous investigations have focused on lacustrine geochemistry [1,2], sedimentology [3], geologic evolution [4-6] and commercial potential [7,8]. Salinity in shallow hypersaline lacustrine environments is extremely sensitive to climatic changes that influence salt concentrations via changes in the evaporation minus precipitation balance, and the hydrologic variability of Kiritimati and Teraina lakes has been attributed to the El Niño Southern Oscillation (ENSO) [1]. ENSO is the largest interannual perturbation to the modern climate system, but the mechanisms that drive its variability remain elusive due to limited monitoring data [9]. Long proxy records of lacustrine hydrology have the potential to elucidate ENSO behavior over centennial to millennial timescales, but require a baseline of modern variability against which past changes can be compared. An extensive description of the lakes of Kiritimati and Teraina that expands upon past work is critically needed to more completely define this baseline variability.

**Figure 2:**
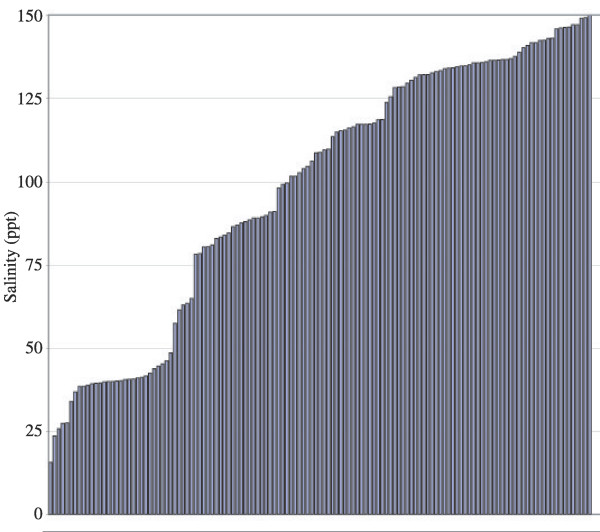
Histogram of salinity values for the lakes survey. Each bar represents an individual lake.

We present data describing the hydrologic variability of Kiritimati and Teraina lakes in June and July of 2005. Because surrounding geology and internal sedimentation influences lacustrine hydrology, and because the salinity of a lake can alter dissolved oxygen and biologic activity, our survey considers chemical (salinity, dissolved oxygen, pH), biological (bacterial mat) and geologic (shore margins, lake sediments) variability. This survey includes fresh (0 ppt), hyposaline (<20 ppt), mesosaline (20–50 ppt) and hypersaline (>50 ppt) lakes [10] (Figure 2), and significantly expands the spatial and temporal coverage of lacustrine monitoring on Kiritimati and Teraina.

### Regional setting

**Figure 3:**
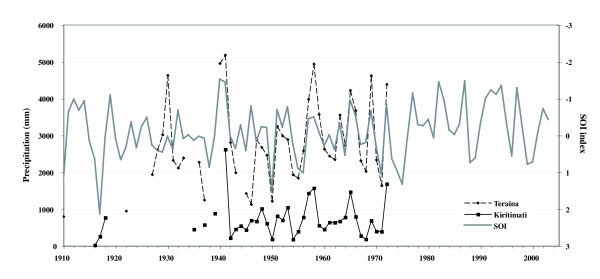
Kiritimati and Teraina rainfall [11] plotted against ENSO variability as defined by the Southern Oscillation Index (SOI). Teraina rainfall January-June, 2005 was measured to be 2464 mm.

Kiritimati (1°52'N, 157°20'W) and Teraina (3°51'N, 159°22'W) Islands are sparsely populated coral atolls separated by ~300 km within the southeast-northwest trending Northern Line Islands of the Republic of Kiribati (Figure 1). Kiritimati is the largest coral atoll in the world with a surface area of ~360 km^2^. The island possesses a uniformly dry climate, and annual precipitation from 1947–1991 averaged 869 mm [6] (Figure 3) with a long-term net rainfall minus evaporation value of -2 mm/day [11]. Despite this evaporative climate, multiple raised sections of the island are underlain by groundwater lenses [12]. The sediments of the island are predominately calcareous and nearly void of any silicate minerals.

**Figure 4:**
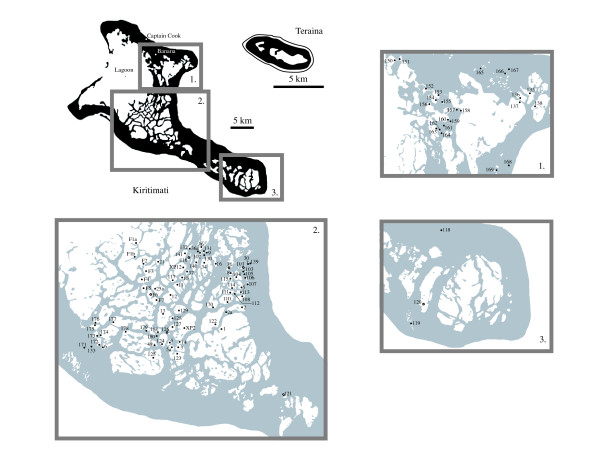
Detailed map of Kiritimati and Teraina islands displaying locations and names of lakes investigated. Open symbols indicate a core was collected. When possible, the closed circle was placed at the location of the survey site.

Approximately a quarter of Kiritimati's surface area is covered by hypersaline lakes, some of which connect to a large lagoon (190 km^2^) in the northwest through an intricate system of channels (Figure 4). This configuration was caused by the isolation of seawater following a mid-Holocene sea level highstand [4,6], and modern lakes represent evaporative basins of trapped seawater [1]. Vegetation surrounding lakes consists of the mangrove *Rhizophora mucronata*, the parasitic climber *Cassytha filiformis*, the grass *Lepturus repens*, and the ironwood *Pemphis acidula*. Other biota includes the land crab *Gecarcoidea natalis*, the brine shrimp *Artemia *and occasionally the milkfish *Chanos chanos*.

In contrast to Kiritimati, Teraina is significantly smaller (14.2 km^2^) with an ovate shape occupied by a large, freshwater lake and a smaller bog in the west (Figure 4). Teraina's freshwater lake is rare in atoll islands, and is maintained by high average annual rainfall of 2903 mm [11] (Figure 3). Lake water is not stratified, but is turbid with high concentrations of floating algal matter that settles to form an organic sedimentary layer above a remnant patch reef [5]. Locally reported fish species include a freshwater trevally (*Caranx sp*.), tilapia (*Oreochromis sp*.), freshwater eel (*Anguilla marmorata*) and freshwater milkfish (*Chanos chanos*). Vegetation is diverse, but is dominated by *Pisonia *forest, while sediments generally consist of organic, phosphatic peat overlying coral sands [5].

Both Kiritimati and Teraina are strongly influenced by ENSO variability as defined by the Southern Oscillation Index (SOI), which measures the difference in pressure between Darwin, Australia and Tahiti. A negative SOI anomaly occurs during an El Niño event, and is marked by increased precipitation on both islands (Figure 3). On Kiritimati, the strong El Niño of 1983 increased the precipitation minus evaporation ratio to +5 mm/day, and decreased lake salinities by 62–90 % [1,13]. Intense rains caused lake stratification and a ~30°C increase in bottom water temperature, as well as CaCO_3 _dissolution [1]. Furthermore, a strong El Niño may augment the typical micro-tidal range by inducing sea-level variations of 30–40 cm [12]. Records are sparser for Teraina, but rainfall records indicate El Niño events may nearly double annual precipitation to over 5000 mm/yr (Figure 3).

## Results

The physical and chemical conditions of lakes are largely governed by the basic hydrologic balance of inputs (i.e. precipitation, groundwater inflow, ocean overwash) and outputs (i.e. evaporation, groundwater seepage), as well as basin morphometry, surrounding geology, biologic activity and climate. Differences in these conditions lead to changes in salinity, pH, DO and other parameters, and the observed variations are described below.

### Kiritimati

#### Lake margins

Lakeshores were generally flat, cemented surfaces ranging from ~5 to hundreds of meters, and consisted of a sequence of four major carbonate facies: paleo-coral, carbonate hardpan, shell-rich cement, and softer sand. Hard coral shores were lowest in the sequence, and could be completely concealed by overlying strata, outcrop at high beach margins, or stretch directly to the lake surface. These thick sequences of paleo-reef often consisted of in situ, growth position *Acropora, Porites *and *Tridacna *species, and typically dropped sharply by approximately a half meter to the lake surface. Hardpan pavements of partially dissolved and reprecipitated carbonates formed broad shores with quasi-hexagonal fractures. These facies typically overlaid paleo-reef sequences, and often connected adjacent lakeshores suggesting periodic connection between adjacent basins. Shell cement margins, commonly overlying coral and hardpan units, were rich in whole pelecypod, gastropod or other mollusk shells, and these units were often indicative of lower salinities. These shores tended to be steep, with aeolian dune deposits, and could be degraded to soft sand by land crab bioturbation or groundwater springs.

Lake fetch and salinity may influence beach morphometry due to the formation of saline foam along the leeward shore of lakes. Foam commonly tumbled from the lake surface and clung to surrounding vegetation where its salinity appeared to stress or kill plants. A larger fetch and higher salinity may create a greater volume of foam detrimental to a wider swath of vegetation, and likely resulted in a broader leeward beach.

### Salinity

**Figure 5:**
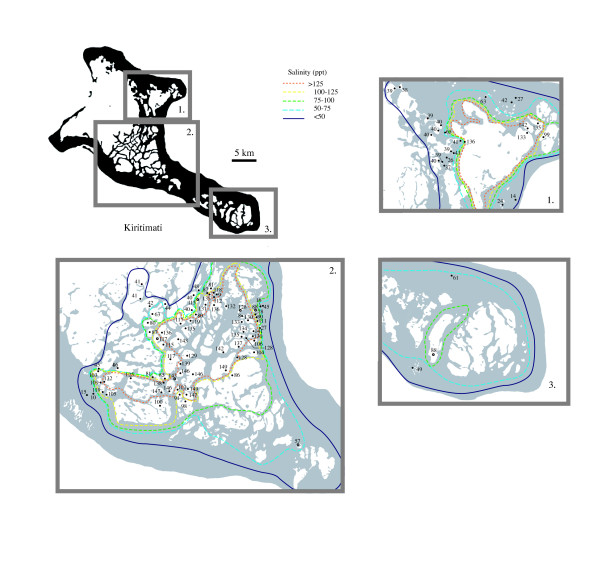
Salinity (ppt) values recorded within Kiritimati lakes with contours indicating a trend toward higher inland salinities.

Lakes varied in salinity from fresh to 150 ppt with a broad range of intermediate values (Figure 5; additional file 1). The lakes of Kiritimati were broadly separated into two categories: those that were part of a connected series, ultimately in communication with the main lagoon, and those that were isolated. Lakes in communication with the lagoon often possessed much lower salinities similar to seawater. In some cases this trend diminished with distance from the lagoon, and some lakes connected to the lagoon through numerous intermediate basins achieved hypersalinity. For example, interconnected F-series lakes (Figure 4, F1a through F7) increased in salinity by nearly 80 ppt toward the center of the island. This series of lakes thus represented a snapshot of the evolution from lagoon to isolated basin as the island emerged from the sea. Exceptions to this trend existed, and lakes in a similar series, such as lakes 152, 153 and 154 showed no salinity increase.

Isolated lakes were more likely to be hypersaline and could exhibit salinities in excess of 150 ppt. Inland basins exhibited the highest salinities, with values dropping to the west toward the lagoon and toward the eastern shore of the island. Salinity reductions were generally small enough to maintain hypersalinity (e.g. lakes 108, 110, 125), but lakes on the east-central shore (e.g. lakes107, 139, 168, 169) dropped more significantly to between 14 and 45 ppt.

Variations with depth were generally small. No salinity stratification was observed through the 6 m water column of lake F6, or the 1.5 m deep lake 120, and values in both basins varied by less than 1 ppt. Lake 180 was the only stratified basin and showed an inverse salinity gradient with 138 ppt surface waters underlain by 124 ppt water at 80 cm. This inverse relation was especially unusual given the lake's positive thermal stratification (32°C at surface, 37°C at 80 cm). The instruments were well calibrated when these data were gathered, and the measurements were replicated to confirm these unusual results.

Occasionally, anthropogenic alteration influenced lake salinities by either artificially creating or destroying connections with other basins. Lakes 174 and 177, once the same basin, were separated by a man-made two-meter berm, and a channel had been constructed to connect lake 177 to the lagoon via lake 176. These alterations were accompanied by a salinity of 46 ppt in lake 177 as opposed to 132 ppt in lake 174. The channel to lake 176 and the lagoon also reduced the salinity of lakes 179 and 181 (81 and 85 ppt respectively) relative to adjacent lake 180 (138 ppt). Conversely, the construction of a road between lakes 175 (103 ppt) and 176 (45 ppt) likely increased the salinity difference between the basins. Because human impact has only existed over the last few decades, the influence of these alterations indicates the lakes of Kiritimati are sensitive to morphological changes, and can respond over decadal or shorter time scales. Interestingly, the series of lakes 172 (119 ppt), 173 (109 ppt) and 175 (103 ppt) have maintained an increasing inland salinity gradient similar to that of the F-series lakes despite having been isolated from the main lagoon.

### pH

**Figure 6:**
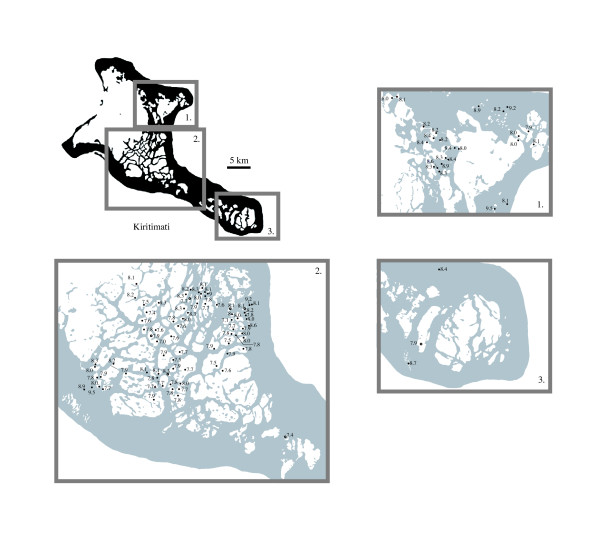
pH values recorded within Kiritimati lakes.

Similar to other hypersaline basins, the lakes of Kiritimati were generally basic, and had pH values ranging from 7 to 10.5 with most values between 7.5 and 8.5 (Figure 6; additional file 1). A weak negative correlation (r = -0.38) existed between pH and salinity with more saline basins typically having lower pH values and fresher lakes having more basic waters. The highest pH values generally occurred in small shallow basins not exceeding a meter of water depth such as lakes 119, 169 and 171.

Variable pH values existed both spatially and temporally, and measurements taken a few meters or hours apart showed a significant offset. This was true of lakes 168 and 169, which were separated by tens of meters, but showed a pH difference of nearly 1.5. Similar degrees of variability were observed on short timescales, and the pH of lake 101 was recorded as 7.6 and 8.4 on successive days without an observed change in salinity. Interestingly, a third survey of the lake two weeks later, and immediately following a rain shower, showed salinity had increased to 102 ppt, but with a negligible pH decrease to 8.3. Lagoon waters, and lakes in close communication with them, exhibited relatively stable pH values slightly higher than seawater, generally between 8.2 and 8.3. No pH variability was observed with depth in either lakes F6 or 120.

Differences in basin shape and the resulting changes in circulation and chemistry showed pH variability consistent with an inverse relationship with salinity. The drop in salinity between lakes 174 and 177 corresponded to a rise in pH from 7.9 in lake 174 to 8.2 in lake 177. Lakes with anthropogenic structures (e.g. lake 126) had pH values 0.4–0.6 higher than adjacent lakes, suggesting physical changes to morphometry could influence pH.

### Dissolved oxygen

DO values, measured on lake bottoms in shallow (~20 cm) nearshore water, varied from 0.61 to 9.46 mg L^-1 ^in absolute terms, and from 17.6 to 190.0% in terms of percent saturation. Lakes exhibited considerable spatial and temporal variability (Additional file 1, Figure 7) though measurements were not taken at a uniform time of day. Generally, isolated hypersaline inland lakes possessed hypoxic DO values from ~2–3.5 mg L^-1 ^and ~35 to 70% saturation while smaller, basins were more likely to have elevated DO exceeding 6 mg L^-1 ^and supersaturated values. Decreasing DO with depth suggested some chemical stratification and values dropped from 2.5 mg L^-1 ^and 63.9% saturation at the surface, to 2.1 mg L^-1 ^and 52.1% saturation at 6 m.

**Figure 7:**
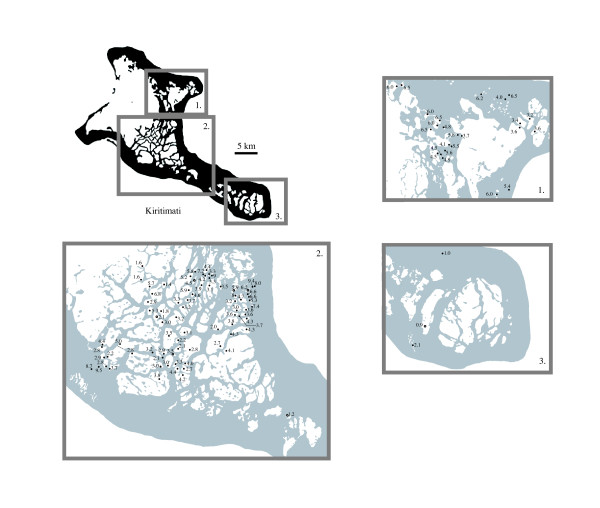
Dissolved oxygen values (mg L^-1^) recorded within Kiritimati lakes.

In general, small, shallow lakes with high surface area to volume ratios had supersaturated DO values, suggesting that photosynthetic mat production and respiration had a major influence on lake DO. Conversely, the presence of fish in many lakes and the potential for greater respiration seemed to play a minor role, but a non-photosynthetic biologic influence on DO cannot be ruled out. High DO values were evident near the small villages of the island where eutrophication was probable, and lakes 150–154, and 156, bordering the settlement of Banana, had near saturated or supersaturated values exceeding 6 mg L^-1^. Lakes 118–120, far removed from settlements, showed low DO values (1.0, 2.1 and 0.9 mg L^-1^; 19.2, 35.4 and 19.6% respectively) as did sites F1a and F1b in the main lagoon (1.6 mg L^-1 ^and 26–27.7% saturation).

### Sediments and bacterial mats

**Figure 8:**
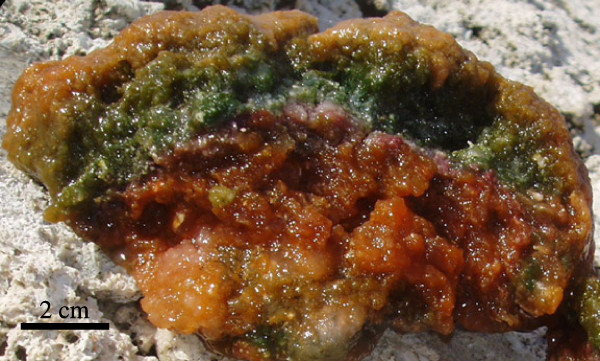
Photograph of a typical mat sequence in which clear chromatic layers are evident.

The surficial sediment of many lakes (additional files 1 and 2) consisted exclusively of gelatinous bacterial mats, a common feature of hypersaline ponds. Mats varied in thickness from a thin veneer of a few millimeters, to almost a half meter. Sharp chromatic stratification in mats was common with regions of mineral precipitate often forming between layers (Figure 8), and lake 128 possessed a unique pink bacterial sequence with an overlying 10 cm thick halite pavement. Mat layers were generally underlain by white-pink carbonate, gypsum or halite precipitate, and varied from very fine clay/silt sized particles to coarse sands, and platy aggregates of mineral, coral or shell rubble. These mineral sequences were more extensive than those between mat layers, reaching thicknesses of over a meter, and are hypothesized to derive from authigenic precipitation, possibly related to ENSO-induced changes in lake chemistry [3].

**Figure 9:**
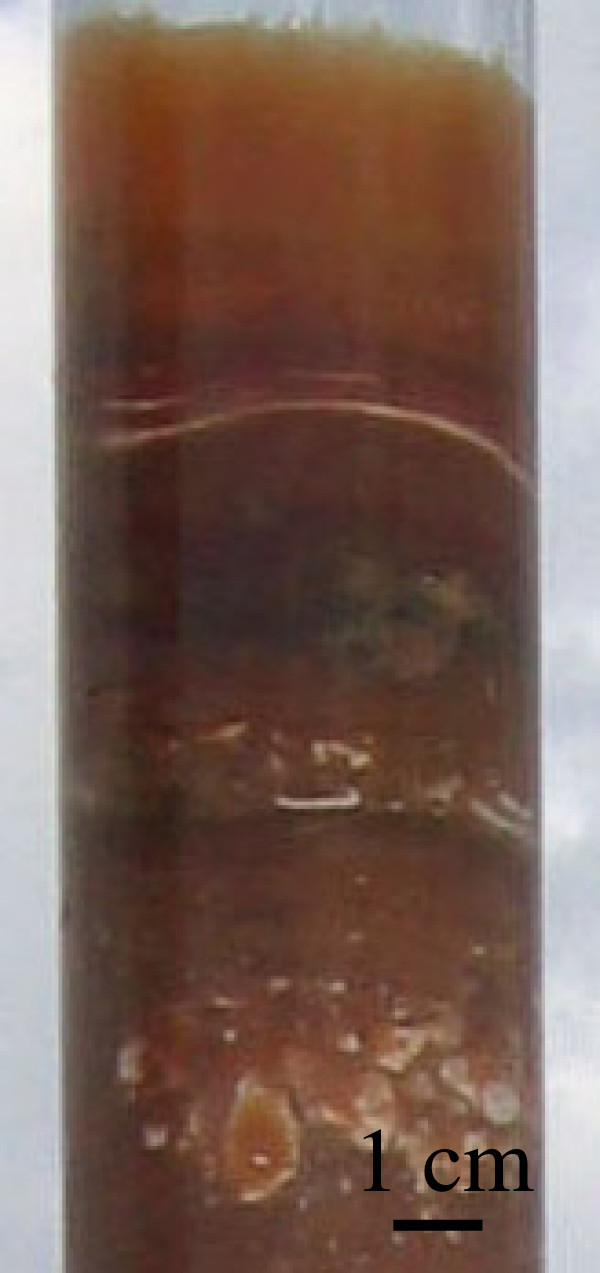
Photograph of sub-millimeter lamination in lake F6 surface sediments.

Previous mat layers were incorporated throughout sediments underlying the upper bacterial mat, and occurred as red, green or brown sub-millimeter laminations (Figure 9). Laminated sequences could extend continuously from the surface or occur more periodically, separated by bulk mineral-mat amalgams. Bulk sections varied in color and texture dependent on the relative proportion of mat to mineral grains. A general trend toward finer grain size with depth was evident in most cores, and this trend terminated in very fine white, silicate-free, carbonate oozes at core bottoms. Total sediment depths varied widely from a few centimeters to 5 meters, and terminated at an impenetrable layer, possibly caused by a coral or hardpan basement.

Some trends were evident between basin morphometry or geography and near-shore sediment characteristics. For example, large lakes with hard coral shores, such as lakes 1, 10, 11 and 12 often possessed little bacterial mat cover and very thin sediment sequences, while smaller shallow basins such as 101 and 113 exhibited the thickest bacterial mat layers and sedimentary sequences exceeding 150 cm. Less saline basins in close communication with the lagoon typically possessed surface sediment dominated by shell cement with very little fine-grained material, and the bottom of these lakes were covered by a thin layer of green algae. Conversely, more saline basins in distant contact with the lagoon, such as lakes F4, F5 and F6, possessed thick sequences of fine white mineralogenic silt. While absent from most basins, fecal matter from fish populations in lake 120 lead to highly flocculated surface sediments.

Anthropogenic activities altered the shallow sediments of select lakes through both the building of infrastructure and aquaculture development. In lake 126, metal stakes remaining from attempts to culture fish, likely contributed to the grey, rather than white-pink color of sediments as well as the unusually thick sequence of green bacterial mat. Similarly, the altered circulation and chemistry of lakes 174, 176 and 177 described above likely caused the unique yellow-green algal film of lake 177 surface sediments. Sediments in the manmade lakes 167, 168 and 171 were often rich in flocculated fish fecies and green algae and may be influenced by the common occurrence of nearby metal debris.

### Teraina

Teraina does not possess the numerous lakes of Kiritimati, but a large peat bog in the western center of the island (Western Peat Bog), a large central fresh-water lake (Washington Lake), and a canal connecting the lake with the sea were surveyed (additional file 3).

### Lake margins

The margins surrounding both the Western Peat Bog, and Lake Washington were heavily vegetated and void of beach sediments. Dense coconut palm jungle gave way abruptly to bog and lake environments, and toppled palms on the Lake Washington shore caused erosion and infilling of the basin. Marsh and peat bog environments were present along the western shore of Lake Washington and exhibited a less sharp transition from jungle to lake surface. The lake basement consisted of *Acropora *and *Tridacna *dominated paleo-reef, which bore a strong resemblance to the sub-aerial reef shore of Kiritimati.

### Salinity

Salinity in both Lake Washington and the canal did not exceed 0.22 ppt, and no stratification was observed in the lake through a three-meter depth range. Salinity in the canal was slightly lower than that of Lake Washington, suggesting one-way water transport from the lake without an influx of marine water. This is consistent with the pressure head that should result from Washington Lake being above sea level.

### pH

Lake Washington was slightly basic, and variations of less than 0.05 pH units reinforced the absence of stratification. pH dropped from 8.4 to 8.1 between two subsequent days due to heavy rains, measuring 64 mm in 12 hours. The uniform pH shift suggested lower pH rainwater was rapidly incorporated throughout Lake Washington, and mixed completely over hourly timescales.

### Dissolved oxygen

Dissolved oxygen was relatively constant, and values ranged from 6.5 and 7.5 mg L^-1 ^with the exception of one portion of the canal that measured 4.7 mg L^-1^. No consistent trend was observed through the 3 meter depth of Lake Washington, and a slight decrease in DO of 0.9 mg L^-1 ^was followed the next day by a mid-depth (1.4 m) increase of ~0.25 mg L^-1^. The large fetch and shallow depth of the lake creates a more hospitable environment for both oxic and photosynthetic primary production, and the absence of stratification suggests wind-driven mixing may be an important factor in Lake Washington's low DO variability.

### Sediments

The surficial sediments of the Western Peat Bog were organic-rich and typically decreased in recognizable plant material to amorphous organic matter with depth until a hard, impenetrable surface was reached at ~50–70 cm. A thicker sediment sequence, reaching up to nine meters was present in Lake Washington and consisted of ~50 cm flocculated green organic rich sediment overlying ~500 cm of flocculated red organic matter, underlain by a sequence of fine white silty clay (additional file 2). White fecal pellets were present throughout the upper green and red organic sequences, and a strong sulfide odor indicated euxinia.

## Discussion

The saline lakes of Kiritimati show remarkable variability, and because local geology and evaporation minus precipitation are relatively uniform across the island, the primary factor influencing salinity distribution is likely location. Increasing salinities in the isolated central-island region suggests less frequent marine inundation, and thus a longer period of evaporation and brine concentration. Sea level can increase by nearly a half meter during El Niño years [12], and it is likely that during these periods many otherwise isolated basins become connected and mix with the lagoon, effectively resetting their evaporative salinity concentration. The slightly higher elevation of inland basins may protect them from higher tidal ranges causing less frequent connection with the lagoon and a higher degree of brine concentration. If evaporation is rapid, a temporary positive thermal stratification, like that of lake 180, may develop owing to greater surficial evaporative cooling.

The permeability of the lake bottoms may also partially govern salinity variability. Fresh groundwater lenses are present in eastern and southwestern Kiritimati as indicated by the presence of freshwater springs and larger palm populations. Subterranean connections between these aquifers and lakes are likely given the permeability of the atoll carbonate sediment, and the lesser hypersalinity or even mesosalinity of some basins may be caused by their proximity to fresh groundwater. Higher salinities may also be the result of reduced lake bottom permeability, and sealing due to bacterial mat growth has been observed in other hypersaline lakes [14].

Variability in DO is likely tied to the combined effects of salinity, basin morphometry, daily sampling time, nutrient loading and bacterial mat symbiosis. Hypersalinity is known to partially inhibit photosynthesis [15] and thus oxygen production, which may be significant given the wide range of salinities present. Additionally, microflora diversity likely increases at lower salinities, which strongly influences the rate of photosynthesis [16]. Given decreasing DO with depth on Kiritimati, surface area to volume ratios may be significant with deeper basins having higher volume to photic zone ratios, and potentially longer mixing times. Natural DO variability also likely follows a radiatively-forced diurnal cycle in production (Table 1). Since DO was not measured at a consistent time, a portion of the observed represents natural daily fluctuations. Furthermore, cyanobacterial photoautotrophs have been shown to be nitrogen limited [17], which suggests higher nutrient concentrations near settlements may lead to greater photosynthetic production, and thus the observed higher DO values. Repiratory or chemical oxidation of H_2_S may also have been responsible for supersaturation in lakes with low water volume to bottom area ratios. A final possible source of variability stems from the complex interconnection between primary production and respiration within bacterial mats. Layers of Kiritimati mat communities are distinct groups with upper surface layers consisting mainly of sheath pigments absorbing 350–450 nm wavelengths, and deeper layers showing pigments characteristic of anoxic purple and green bacteria [18,19]. These communities are likely consortia; a characteristic which permits the cycling of potentially limiting nutrients and which often forms closely spaced micro-niches [20,21]. Complex symbiosis exists with respect to oxygen utilization in bacterial mats with regions of oxygen consumption and production known to occur in close proximity [22]. Such tight coupling makes these communities especially susceptible to small environmental perturbations, and may be responsible, at least in part, for the wide variations observed in lake DO.

**Table 1:** Diel variability of chemical properties within lake F6

Lake Number	Sampling date	Time	Temp (1/4C)	Conductivity (μS)	Resistance (Ω)	Salinity (ppt)	DO (% sat.)	DO (mg L^-1^)	pH
F6	6/22/05	9:35	29.58	147121	6.249	116.47	36.3	1.45	7.95
F6	6/22/05	10:00	30.73			117.17	50.1	1.98	7.98
F6	6/22/05	10:11	31.03			117.46	52.2	1.99	7.98
F6	6/22/05	10:34	31.51			117.70	52.0	2.00	7.98
F6	6/22/05	11:17	32.38			117.93	65.6	2.43	7.97
F6	6/22/05	15:36	33.61	151903	5.6536	121.12	49.4	1.81	7.93

Both inorganic and biological processes influence the tendency for more saline basins to have lower pH values. As evaporation proceeds, calcite, gypsum, halite and various chlorides will precipitate in that order, and the most hypersaline lakes likely exhibit losses of Ca^2+^, Na^+^, and K^+ ^as magnesium becomes the dominant cation [23]. High carbonate deposition is known to occur on Kiritimati relative to other evaporative salinas, potentially because of cyanobacterial organomineralization [3]. Calcite precipitation reduces lake water carbonate ion (CO_3_^2-^) concentrations, which removes alkalinity, reduces carbonate buffer strength and lowers the lake pH. A similar mechanism likely explains more acidic conditions in other evaporite basins with salinities in excess of 80 ppt [24]. The potential of lower photosynthetic rates at higher salinities noted above may also partially explain the inverse relationship between salinity and pH. All other factors being equal, lower photosynthetic rates would remove less aqueous carbon dioxide from lake water, thus leaving a higher carbonic acid concentration. This is consistent with the weak positive correlation between pH and DO that would be expected from dependence on mat photosynthesis and respiration. Production at the bottom of mat sequences, where H_2_S, not H_s_O is the terminal electron receptor, may also influence pH due to the production of SO_4_^2- ^or elemental S rather than oxygen.

Comparing our observations with previous accounts of lacustrine variability provides insight into longer-term salinity variability and its connection with climate (Table 2). Valencia [4] reports lakes 131 and 13 to have salinities in excess of 300 ppt during the spring and autumn of 1970; more than twice the 130 and 117 ppt values measured in this study. Similarly, mesosaline (36–41 ppt) rather than fresh springs were reported to enter inland hypersaline lakes in 1970, suggesting more saline groundwater and drier conditions [4]. In the strong El Niño year of 1983, Schoonmaker [1], found surface salinities significantly lower than 2005 values (e.g. 40.5, 55 and 41 ppt in lakes 2, 3 and 5 respectively), but often comparable at ~2 m (164 and 102 in lakes 3 and 5 respectively). While some of variability may be due to natural seasonal fluctuations, the observed pattern suggests conditions similar to those observed in 2005 persisted prior to El Niño-related surficial freshening. Surface salinities in 1984 were comparable to those measured in 2005 as well as those measured at 2 m in 1983 (e.g. 127 ppt in 1984 lake 3 surface waters), indicating less prominent stratification in the year following an El Niño. The similarities between 1983 pre-El Niño bottom salinities and moderately recovered 1984 salinities, suggest 2005 values may be typical of average salinities. Higher 1970 salinities appear elevated from these proposed background values, possibly due intra-annual variability or to a La Niña-like mean climate state with fewer strong El Niño events in the twenty preceding years.

**Table 2:** Summary of previously reported chemical and physical properties of Kiritimati and Teraina lakes

Lake Number	Sampling date	Temp (1/4C)	Salinity (ppt)	Depth (m)	pH	Max Water (cm)	max sed (cm)	Sediment description	Shore description	Other information
1	1983	30.0	49.00	surface	8.12					Schoonmaker et al., 1985
2	1983	33.4	40.50	surface	8.08					Schoonmaker et al., 1985
3	1983	30.2	55.00	surface	8.14					Schoonmaker et al., 1985
3	1983	50.0	164.00	~2	7.26					Schoonmaker et al., 1985
4	1983	28.7	28.10	surface	8.28					Schoonmaker et al., 1985
5	1983	29.5	41.20	surface	8.00					Schoonmaker et al., 1985
5	1983	44.6	102.00	~2	7.31					Schoonmaker et al., 1985
6	1983	30.0	21.00	surface	8.29					Schoonmaker et al., 1985
7	1983	26.7	1.50	surface	8.59					Schoonmaker et al., 1985
8	1983	26.9	7.60	surface	8.37					Schoonmaker et al., 1985
9	1983	28.2	35.30	surface	8.58					Schoonmaker et al., 1985
9	1983	37.0	247.00	~2	6.72					Schoonmaker et al., 1985
13	1983	28.9	24.70	surface	8.89					Schoonmaker et al., 1985
10	1983	28.3	40.10	surface	8.47					Schoonmaker et al., 1985
10	1983	28.3	40.20	1.0	8.46					Schoonmaker et al., 1985
10	1983	28.3	40.10	2.0	8.45					Schoonmaker et al., 1985
10	1983	28.5	40.50	3.0	8.46					Schoonmaker et al., 1985
10	1983	31.0	44.70	3.2	8.39					Schoonmaker et al., 1985
10	1983	54.0	88.00	3.4	8.04					Schoonmaker et al., 1985
10	1983	58.5	233.00	4.0	7.15					Schoonmaker et al., 1985
10	1983	59.5	232.00	4.5	7.19					Schoonmaker et al., 1985
10	1983	60.7	239.00	5.0	7.10					Schoonmaker et al., 1985
										
1	1984	30.0	78.38	surface	7.8					Schoonmaker et al., 1985
2	1984	28.0	66.21	surface	8.09					Schoonmaker et al., 1985
3	1984	29.3	126.52	surface	8.52					Schoonmaker et al., 1985
7	1984	28.5	25.31	surface	8.48					Schoonmaker et al., 1985
12	1984	28.8	117.54	surface	8.4					Schoonmaker et al., 1985
13	1984	29.5	39.19	surface	8.13					Schoonmaker et al., 1985
17	1984	31.0	22.42	surface	8.75					Schoonmaker et al., 1985
17	1984	31.0	22.23	surface	8.61					Schoonmaker et al., 1985
18	1984	30.3	14.77	surface	8.15					Schoonmaker et al., 1985
19	1984	30.0	34.24	surface	8.12					Schoonmaker et al., 1985
20	1984	29.6	41.07	surface	8.28					Schoonmaker et al., 1985
21	1984	28.6	106.99	surface	8.14					Schoonmaker et al., 1985
22	1984	28.1	75.85	surface	8.34					Schoonmaker et al., 1985
23	1984	28.9	42.03	surface	8.15					Schoonmaker et al., 1985
24	1984	29.0	91.78	surface	8.29					Schoonmaker et al., 1985
25	1984	28.5	2.00	surface	9.07					Schoonmaker et al., 1985
10	1984	32.5	111.87	surface	8.2					Schoonmaker et al., 1985
10	1984	28.6	140.76	~0.5	8.16					Schoonmaker et al., 1985
10	1984	27.2	156.61	~2.5	8.08					Schoonmaker et al., 1985
10	1984	37.0	198.71	~3	7.84					Schoonmaker et al., 1985
10	1984	44.0	235.48	~4	7.43					Schoonmaker et al., 1985
10	1984	29.4	142.63	surface	8.07					Schoonmaker et al., 1985
10	1984	29.0	142.14	0.5	8.1					Schoonmaker et al., 1985
10	1984	29.0	142.18	1.0	8.1					Schoonmaker et al., 1985
10	1984	29.1	142.26	1.5	8.09					Schoonmaker et al., 1985
10	1984	29.1	142.25	2.0	8.08					Schoonmaker et al., 1985
10	1984	30.7	149.30	2.5	8.06					Schoonmaker et al., 1985
10	1984	37.5	200.39	3.0	7.86					Schoonmaker et al., 1985
10	1984	41.6	219.59	3.5	7.58					Schoonmaker et al., 1985
10	1984	43.5	233.08	4.0	7.47					Schoonmaker et al., 1985
10	1984	46.4	238.08	4.4	7.38					Schoonmaker et al., 1985
										
16a	1970								Less saline springs (36–41 ppt)	Valencia, 1977; original lake number
131	1970		300+							Valencia, 1977; author's lake 19d
19e	1970		300+							Valencia, 1977; original lake number
27a	1970								Less saline springs (36–41 ppt)	Valencia, 1977; original lake number
13	1970		300+					5 cm gypsum halite crust overlying red mat		Valencia, 1977; author's lake 33a
Western Peat Bog							70	Sand, peat and clay, dated to 1060 +/- 100 radiocarbon years		Wester et al., 1992
Lake Washington						10		Significant remnant reef		Wester et al., 1992

The variability between Kiritimati and Teraina hint at longer term climate changes. Similar underlying reef structures and the observation that modern Kiritimati lake levels are below sea level, while Lake Washington is above sea level suggests Teraina and Kiritimati paleo-reefs are derived from the same sea level highstand. Hypersaline lakes do not presently exist on Teraina because the island underlies the global precipitation band that occurs at the convergence of northern and southern hemisphere trade winds. Known as the Intertropical Convergence Zone (ITCZ), this region of high precipitation does not presently impact Kiritimati. The red silt sequence overlying white silty clay in Lake Washington bears resemblance to the sedimentary sequences of many Kiritimati cores, and it is tempting to propose that Lake Washington once had a drier climate and hypersaline lakes that supported bacterial mats. Drier conditions would be consistent with suggestions that the ITCZ occupied a more northerly position during the early Holocene [25-27]. Thus, when sea level fell, isolating lakes on Teraina, the ITCZ may have had little influence, and allowed hypersaline lakes and bacterial mats to form. As the ITCZ migrated south over the next few thousand years, the hypersaline ponds may have been overwhelmed by precipitation and transformed into a freshwater environment.

## Conclusion

This survey indicates the lakes and bogs of Kiritimati and Teraina islands possess a large degree of natural variability on timescales of days, decades and possibly millennia. Chemical variability within lake series of progressively more isolated basins may serve as a modern parallel to conditions spanning the emergence of Kiritimati, effectively permitting paleonvironmental reconstructions through time. Within the context of previous work, and given the relatively neutral ENSO state of 2005, these observations may be typical of baseline variability. Further monitoring and high-resolution proxy reconstructions of past lake hydrology could provide paleoclimatological information critical to understanding

ENSO variability.

## Methods

The chemistry and surface sediment of over one hundred lakes were sampled during a five-week period spanning June and July 2005. Salinity, pH and DO were measured using a portable YSI Sonde 6600 connected to a YSI 650 MDS data logger. It was not possible to sample all lakes at uniform times of day. Calibrations for salinity, pH, and DO were performed regularly using local seawater as a 35 ppt endmember for salinity, pH 4,7 and 10 buffer solutions, and water saturated air with temperature correction respectively. The response of the instrument was repeatedly checked using serial dilutions of the most concentrated samples, and was found to be linear to 140 ppt with values from 140–150 ppt underestimating salinity by ~6%. Surficial lake sediments were documented with digital photography, and their depth was assessed using a stainless steel probe. Grabs of surface sediments were recovered from all lakes, and homogenized the upper ~5 cm of the sedimentary sequence. Longer records of recent sedimentation were collected in select lakes using either a Russian peat corer (up to 50 cm) or rod-operated piston corer (up to 150 cm) with a clear PVC core tube. Both techniques preserved laminations and permitted sediments to be visually inspected and described. Longer piston cores, up to 9 m were also collected in select lakes, and a preliminary evaluation of stratigraphy could be made from exposed sediments at either end of 100 cm aluminum core tubes.

## Competing interests

The author(s) declare that they have no competing interests.

## Authors' contributions

CS conducted field work, compilation, analysis and reporting. MM conducted field work, and served as an advisor to the project. RS conducted field work, and served as an advisor to the project. JS conducted field work, and served as an advisor to the project.

## Supplementary Material

Additional File 1 Physical and chemical characteristics of all Kiritimati lakes surveyed Click here for file

Additional File 2 Description of surficial sediment cores collected on both Kiritimati and Teraina Islands Click here for file

Additional File 3 Physical and chemical characteristics of Washington Lake and drainage canal on Teraina Click here for file
