# Supplementary material for: A physico-chemical survey of inland lakes and saline ponds: Christmas Island (Kiritimati) and Washington (Teraina) Islands, Republic of Kiribati
Source: Saline Syst. 2006 Jul 3;2:8. doi: 10.1186/1746-1448-2-8 (PMC1533837; doi:10.1186/1746-1448-2-8)
Supplement: Supplementary file 1 [file 1746-1448-2-8-S3.doc]

Table 3

**Lake Number**	Sampling date	Time	Temp (ºC)	Conductivity (mS)	Resistance (W)	Salinity (ppt)	DO (% sat.)	DO (mg L^{-1})	Depth (m)	pH	max water (cm)	max sed (cm)	Sediment description	Shore description	Other information
Wash-ington Lake	7/7/05	12:53	29.13	454	2042	0.22	96.7	7.41	0.187	8.35	~370	700-900	~50 cm green flocuated silt overlying ~500 cm red flocuated silt with fecal matter in both units. Fine white, likely calcareous, silt/clay below 500 cm	Jungle soil and peat with sharp, ~50 cm drop to lake surface	Surface sample. Secchi depth of 2.81 m
Wash-ington Lake	7/7/05		29.16	454	2042	0.22	91.1	6.98	2.357	8.36	~370	700-900	~50 cm green flocuated silt overlying ~500 cm red flocuated silt with fecal matter in both units. Fine white, likely calcareous, silt/clay below 500 cm	Jungle soil and peat with sharp, ~50 cm drop to lake surface	Mid-depth sample. Secchi depth of 2.81 m
Wash-ington Lake	7/7/05		29.14	454	2042	0.22	90.2	6.91	2.577	8.37	~370	700-900	~50 cm green flocuated silt overlying ~500 cm red flocuated silt with fecal matter in both units. Fine white, likely calcareous, silt/clay below 500 cm	Jungle soil and peat with sharp, ~50 cm drop to lake surface	Mid-depth sample. Secchi depth of 2.81 m
Wash-ington Lake	7/7/05		29.11	453	2044	0.22	85.2	6.53	3.097	8.33	~370	700-900	~50 cm green flocuated silt overlying ~500 cm red flocuated silt with fecal matter in both units. Fine white, likely calcareous, silt/clay below 500 cm	Jungle soil and peat with sharp, ~50 cm drop to lake surface	Bottom sample. Secchi depth of 2.81 m
Wash-ington Lake	7/7/05		29.13	454	2043	0.22	84.6	6.49	3.008	8.33	~370	700-900	~50 cm green flocuated silt overlying ~500 cm red flocuated silt with fecal matter in both units. Fine white, likely calcareous, silt/clay below 500 cm	Jungle soil and peat with sharp, ~50 cm drop to lake surface	Bottom sample. Secchi depth of 2.81 m
Wash-ington Lake	7/8/05	12:24	28.37	455	2062.7	0.22	89.7	6.97	0.71	8.11	~370	700-900	~50 cm green flocuated silt overlying ~500 cm red flocuated silt with fecal matter in both units. Fine white, likely calcareous, silt/clay below 500 cm	Jungle soil and peat with sharp, ~50 cm drop to lake surface	Surface sample. Secchi depth of 2.81 m
Wash-ington Lake	7/8/05	12:22	28.37	458	2050.6	0.22	92.9	7.22	1.372	8.07	~370	700-900	~50 cm green flocuated silt overlying ~500 cm red flocuated silt with fecal matter in both units. Fine white, likely calcareous, silt/clay below 500 cm	Jungle soil and peat with sharp, ~50 cm drop to lake surface	Mid-depth sample. Secchi depth of 2.81 m
Wash-ington Lake	7/8/05	12:23	28.4	457	2055.2	0.22	90.6	7.04	2.975	8.11	~370	700-900	~50 cm green flocuated silt overlying ~500 cm red flocuated silt with fecal matter in both units. Fine white, likely calcareous, silt/clay below 500 cm	Jungle soil and peat with sharp, ~50 cm drop to lake surface	Bottom sample. Secchi depth of 2.81 m
Canal	7/7/05	16:07	27.78	203	4686.7	0.09	85	6.67	0.337	7.99	~200			Jungle soil with regions of peaty marsh	
Canal at Wash-ington Lake	7/8/05	8:59	26.5	335	2898	0.16	53.3	4.69	0.202	7.59				Jungle soil with regions of peaty marsh	Measured where the canal enters Lake Washington
